# Comparing the Safety and Effectiveness of Ketamine Versus Benzodiazepine/Opioid Combination for Procedural Sedation in Emergency Medicine: A Comprehensive Review and Meta-Analysis

**DOI:** 10.7759/cureus.36742

**Published:** 2023-03-27

**Authors:** Hany A Zaki, Tarek Ibrahim, Ahmed Osman, Wael Abdelrehem Elnabawy, Amr Gebril, Ahmed H Hamdi, Eslam Hussein Mohamed

**Affiliations:** 1 Emergency Medicine Department, Hamad Medical Corporation, Doha, QAT; 2 Emergency Department, Zagazig University, Zagazig, EGY; 3 Emergency Medicine, Yasclinic Hospital, Abu Dhabi, ARE; 4 Emergency Department, Hamad Medical Corporation, Doha, QAT; 5 Emergency Medicine, NMC Hospital, Abu Dhabi, ARE

**Keywords:** systematic review and meta-analysis, emergency department, psa, procedural sedation and analgesia, opioids, benzodiazepines

## Abstract

Procedural sedation is essential in the ED to conduct painful procedures effectively. Ketamine and benzodiazepines/opioids are commonly used, with ketamine providing adequate analgesia and preserving airway muscle tone. However, ketamine is associated with adverse effects while benzodiazepines/opioids can lead to respiratory depression. This study compares the safety and efficacy of ketamine and midazolam/fentanyl.

Two search methods were used to identify studies related to our topic, including a database search and a manual search involving screening reference lists of articles retrieved by the database search. A methodological quality appraisal was conducted on the articles suitable for inclusion using Cochrane’s risk of bias tool in the Review Manager software (Review Manager (RevMan) (Computer program). Version 5.4, The Cochrane Collaboration, 2020). Moreover, pooled analysis was performed using the Review manager software.

The study analyzed 1366 articles, of which seven were included for analysis. Pooled data showed that ketamine and midazolam/fentanyl had similar effects on pain scores during procedures and sedation depth measured by the University of Michigan sedation scale. However, the Modified Ramsay Sedation Score showed significantly more profound sedation in the ketamine group. The only significant adverse events were vomiting and nausea, which had a higher incidence in the ketamine group.

Our data suggest that ketamine is as effective as the midazolam/fentanyl combination for procedural sedation but is associated with higher incidences of adverse events. Therefore, midazolam/fentanyl can be recommended for procedural sedation in the ED. However, it should be provided in the presence of a physician comfortable with airway management due to high incidences of oxygen desaturation.

## Introduction and background

Procedural sedation is a vital technique that helps emergency physicians (EPs) conduct painful procedures humanely and timely in the emergency department (ED). However, sedation might result in serious adverse events without proper training, monitoring of vital signs, and quality assurance. A 1995 study by Quine reported a mortality rate of approximately one in every 2000 patients sedated for gastroscopy in the United Kingdom (UK) and found that only 40% of the patients had monitoring of oxygen saturation during the procedure [1]. Since then, multiple studies have proved that procedural sedation is safe and deaths are eradicated when the current sedation guidelines are adhered to [2,3].

Various agents, including benzodiazepines, opioids, ketamine, nitrous oxide, etomidate, and propofol, have been used for procedural sedation. Ketamine is a phencyclidine derivative that offers a unique dissociative anesthetics state [4]. It usually provides excellent analgesia and amnesia while favorably preserving airway muscle tone, airway reflexes, and spontaneous respiration [4,5]. It can be administered in various ways, including intravenously (IV) and intramuscularly (IM). The IV administration (1-2 mg/kg) over 30-60 seconds usually allows fast recovery and evades transient apnea that is likely to manifest in a rapid push.

On the other hand, IM administration (4-5 mg/kg) is usually used when IV administration is challenging; however, this technique is subject to an increased risk of emesis [6]. Despite the unique dissociative aspect of ketamine, it is subject to several adverse effects, with the most notorious impact being the emergence of reactions that include delirium, agitation, and combativeness. Traditionally, these effects are minimized by pre-emptively administering benzodiazepines. Ketamine has also been associated with a 0.3% occurrence of idiosyncratic laryngospasm and a possible increase in intracranial pressure; thus, it should be used cautiously in patients with existing airway or intracranial disease [7].

On the other hand, opioids are combined with benzodiazepines to accentuate risks. The most popular benzodiazepine/opioids combination in procedural sedation is the combination of midazolam with fentanyl. A study by Kennedy and colleagues showed that the combination resulted in complete amnesia in 85% of the patients and resulted in a low rate of nausea and vomiting (9%) and hypotension (6.2%) [8]. However, this combination regimen is associated with an increased risk of respiratory depression. Cevik and colleagues reported a very high hypoxia incidence (76.7%), requiring positive-pressure ventilation in one of the patients [9]. The midazolam-fentanyl combination has also been associated with higher pain, anxiety, and distress scores than other common drug combinations [8,9].

To our knowledge, no systematic review has been conducted to compare ketamine to a combination of benzodiazepines and opioids in procedural sedation. Therefore, the current study will compare the efficacy and safety of ketamine with the most common benzodiazepine/opioid combination (midazolam/fentanyl) and evaluate which regimen should be considered for procedural sedation.

## Review

Methodology

Protocol and Registration

We prepared this article by observing and following the Cochrane Collaboration guidelines. The results were reported as per the PRISMA (Preferred Reporting Items for Systematic Reviews and Meta-Analyses) guidelines.

Eligibility Criteria

One reviewer created the criteria to include and exclude articles from the current review. The inclusion criteria were outlined as follows: Studies that directly compared the efficacy or safety of ketamine with any combination of benzodiazepine with opioids. Observational and randomized trials published in English. This criterion was vital since it helped evade direct translations of scientific terms that would otherwise tamper with the scientific research of the present study. And studies in which procedural sedation was provided in an emergency setting.

On the other hand, the exclusion criteria were as follows: Studies designed as either letters to the editor, guidelines, abstracts without full articles, systematic reviews, and case reports. Studies compared ketamine combined with other sedatives to a combination of benzodiazepines with opioids or compared ketamine to individual benzodiazepines or opioids. And studies that generally evaluated the safety of ketamine and benzodiazepines/opioids without specifying the various complications associated with each sedation agent.

Literature Search

Two search methods were used to identify studies related to our topic, a database search, and a manual search. The database search involved utilizing a well-defined search strategy on the following electronic databases; PubMed, ScienceDirect, Medline, Google Scholar, and Scopus. This search strategy was as follows; (“benzodiazepines” AND “Opioids” OR “Benzodiazepines/opioids” OR “fentanyl/midazolam” OR “fentanyl/diazepam” OR “morphine/midazolam” OR “morphine/diazepam”) AND (“procedural sedation” OR “PSA”) AND (“Emergency setting” OR “Emergency department” OR “Emergency room”). On the other hand, the manual search involved perusing the reference lists of studies identified from the electronic databases for additional studies. We avoided retrieving close or exact duplicates and gray literature during the search. This specification was important for our research because these articles would have undermined our scientific research.

Quality Assessment

For this study, the sedation depth was analyzed using two sedation score scales, including the University of Michigan sedation scale (UMSS) and the Modified Ramsay sedation score (MRSS) [10,11]. In addition, oxygen desaturation was considered an adverse event if patients required termination of the procedure and/or intervention to resolve the condition.

**Figure 1:**
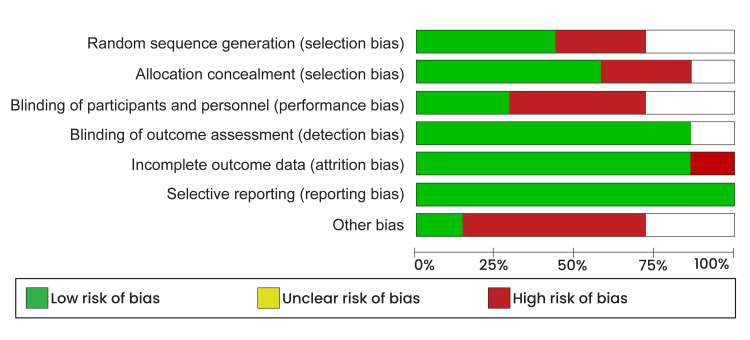
Risk of bias graph

The current study was designed as an interventional review; therefore, the Cochrane Risk of bias tool provided in the Review Manager software (RevMan 5.4.1; (Review Manager (RevMan) (Computer program). Version 5.4, The Cochrane Collaboration, 2020)) was used for the methodological quality assessment. This assessment method employed four criteria: selection, attrition, performance, and reporting bias. A low risk of bias was given a green color when the assessment criteria were fully answered while a high risk of bias was given a red color and used when the criteria were not answered. On the other hand, the unclear risk of bias was not posted in color and was used when the reviewers could not make a clear judgment due to few details; the risk of bias graph is shown in Figure 1.

**Figure 2:**
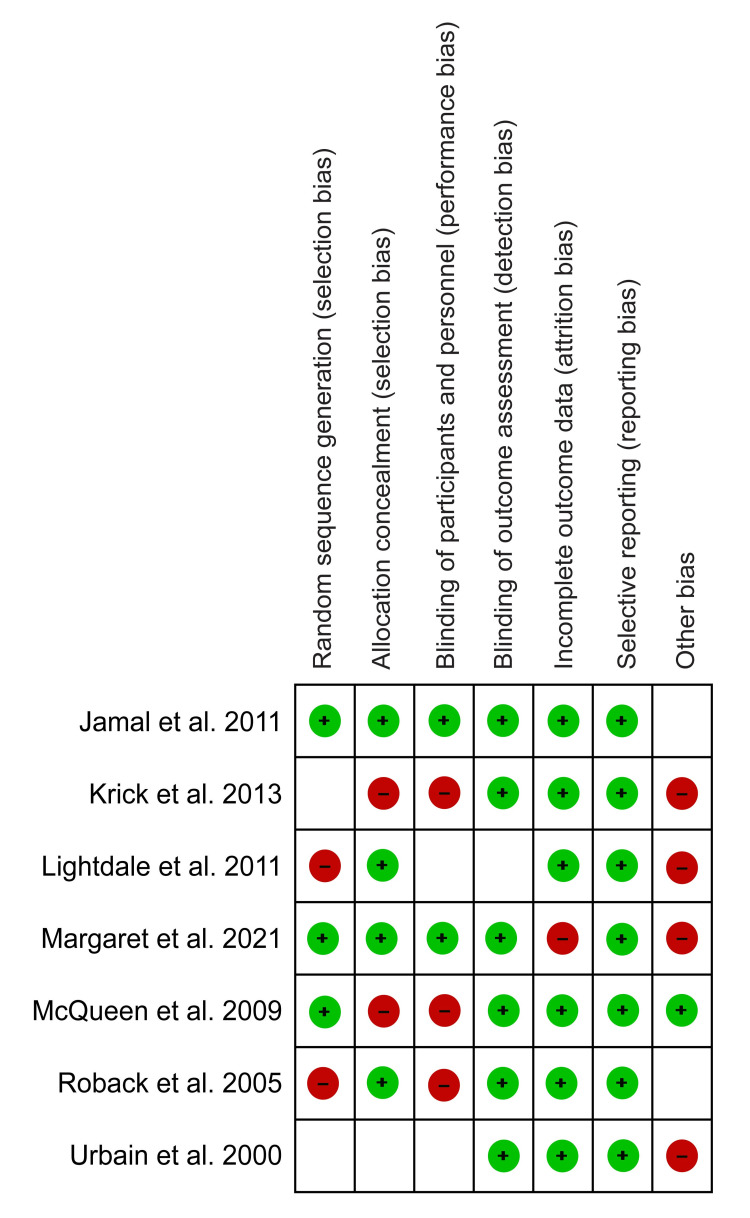
Risk of bias summary Jamal et al., 2011 [12], Roback et al., 2005 [13], McQueen et al., 2009 [14], Urbain et al., 2000 [15], Lightdale et al., 2011 [16], Krick et al., 2013 [17], Margaret et al., 2021 [18]

Figure 2 shows the risk of bias summary.

Data Extraction and Definitions

The data extraction process was done by two reviewers, who then summarized the data into a table. The data retrieved from each study included: author ID (surname of the first author and the year the study was first published), participants’ characteristics (sample size, mean age, and gender distribution), location of the analysis, study design, ketamine and benzodiazepine/opioid dosages, the procedure(s) performed after sedation, and the main outcomes. The preliminary results of this study were the adverse events associated with sedation regimens, sedation depth, and pain scores during the procedures. In the event of discrepancies, the two reviewers resolved their differences through an interactive discussion or by consulting a third reviewer who acted as an arbitrator.

Data Synthesis

The Review Manager software (RevMan 5.4.1) was used to analyze the differences in the rate of adverse events, sedation depth, and pain score reductions. Data about adverse events was dichotomous; therefore, the difference was calculated using the simple odds ratio (OR) and a 95% confidence interval (CI). On the other hand, data regarding pain scores and sedation depth was continuous; therefore, the difference was calculated using the standard mean difference (SMD) and a 95% CI. Our analysis also involved computation of heterogeneity between the studies, of which values between 0 and 49, 50 and 69, and 70 and 100 were regarded as low, moderate, and high, respectively. All the pooled effect sizes were then presented in forest plots, of which a significance level of less than 0.05 was considered statistically significant.

Results

Study Selection

**Figure 3:**
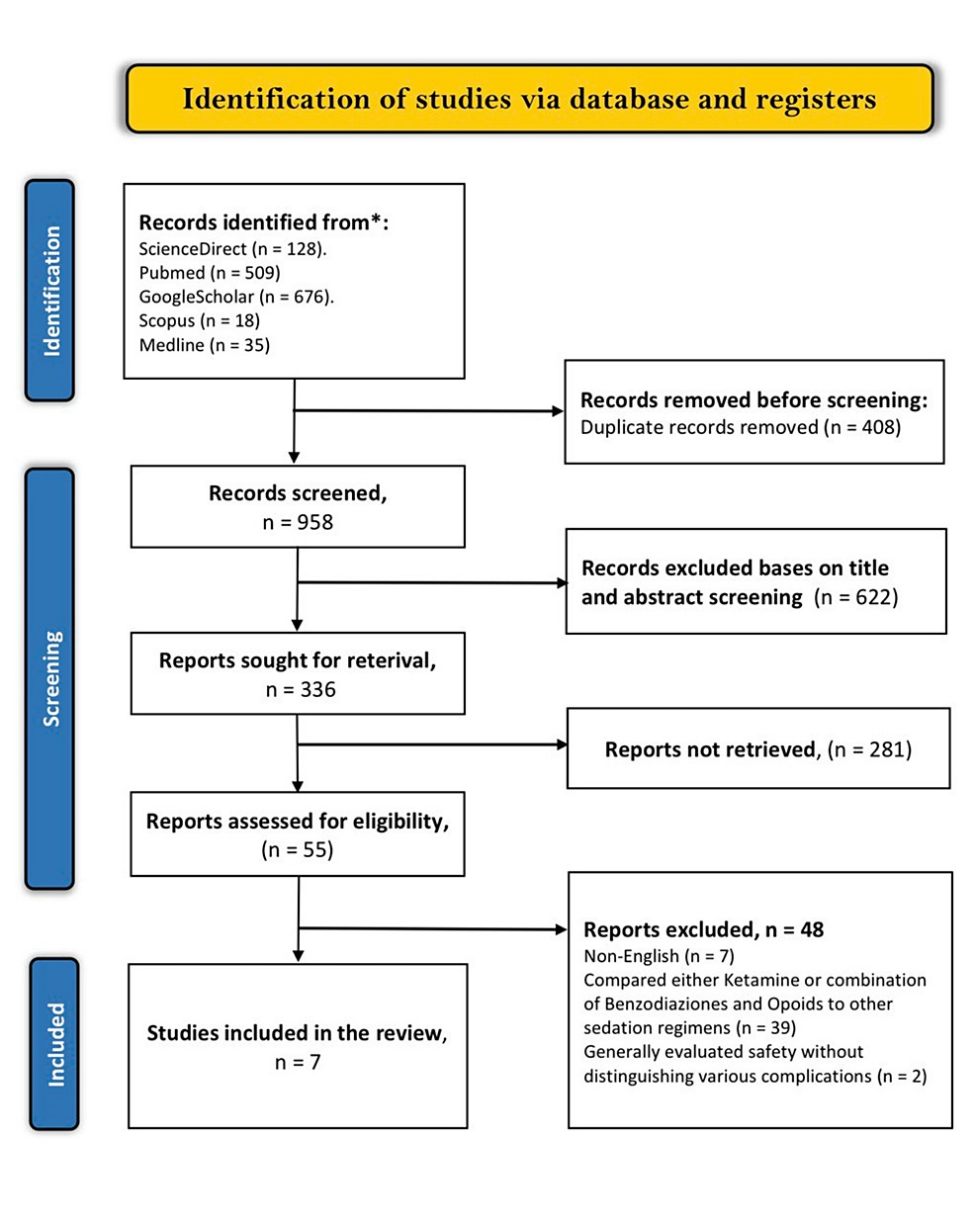
PRISMA flow diagram of the literature search results PRISMA: Preferred Reporting Items for Systematic Reviews and Meta-Analyses

Using the database search method, we attained 1366 articles with the keywords specified in the search strategy. We analyzed these articles to identify duplicates and found that 408 were either close or exact duplicates. The duplicates were excluded from the study, and the remaining articles had their papers and abstracts screened, of which 622 were excluded based on this screening process. Out of the 336 remaining articles, we did not retrieve 281 because they were either ongoing trials, abstracts without full evidence, letters to the editor, guidelines, case reports, or systematic reviews. At the end of the study selection process, we only identified seven articles suitable for analysis in this review. The other 48 articles were unsuitable due to the following reasons; seven were published in different languages, 39 compared ketamine combined with other sedatives to a combination of benzodiazepines with opioids or compared ketamine to individual benzodiazepines or opioids, and two evaluated the safety of ketamine and benzodiazepines/opioids but did not specify the various complications associated with each sedation agents. A summarized literature selection criteria are shown in the PRISMA diagram below (Figure 3).

Study characteristics are described in Table 1.

**Table 1:** Study characteristics Jamal et al., 2011 [12], Roback et al., 2005 [13], McQueen et al., 2009 [14], Urbain et al., 2000 [15], Lightdale et al., 2011 [16], Krick et al., 2013 [17], Margaret et al., 2021 [18]

Author ID	Study Design	Location	Participants’ characteristics	Ketamine Dose	Benzodiazepines/opioids Dose	Procedures	Main Outcomes
Jamal et al., 2011 [12]	RCT	Malaysia	41 patients (32 males and 9 females)	An IV dose of 0.5 mg/kg titrated every 3 minutes up to a maximum dose of 2 mg/kg	A single dose of IV fentanyl at a 1 mcg/kg dose followed by IV 0.05 mg/kg midazolam titrated every 3 minutes up to a maximum dose of 7.5 mg	Fracture and Joint reductions	The procedure was successful in 95.7% of patients that received Midazolam/fentanyl and 94.4% of patients that received IV ketamine. The two groups did not show a significant difference in pain scores after sedation (1 vs. 3, for Midazolam/fentanyl and Ketamine group, respectively.
Roback et al., 2005 [13]	Retrospective cohort study	United States	2500 patients (1511 males and 989 females; median age: 6.7 years (19 days – 32 years)).	NR	NR	Fracture reduction, laceration repair, lumbar puncture, and Imaging	91 patients that received ketamine alone experienced respiratory adverse events, while 151 vomited after sedation. Of the 336 patients that received midazolam/fentanyl, 65 experienced respiratory adverse events, and 6 vomited. Apnea and laryngospasm were recorded in 11 and 1 patients that received ketamine alone, respectively.
McQueen et al., 2009 [14]	Prospective observational study.	United States	554 patients (213 females and 341 males; mean age: 7.5 + 4.5 years)	NR	NR	Fracture reduction and laceration repair	Vomiting was reported in 25 patients in the ketamine group and 4 in the midazolam/fentanyl group.
Urbain et al., 2000 [15]	Retrospective study	Canada	167 patients (103 males and 64 females; 6 months to 16 years).	IV 2.0 – 5.0 mg/kg in 129 patients and IM 0.5 – 1.0 mg/kg in 5 patients	IV 0.05 – 0.1 mg/kg of midazolam followed by IV 2.0 – 5.0 mg/kg of fentanyl	Orthopedic manipulation, wound repair, and foreign body removal	Of the 138 patients that received ketamine, 17 vomited, while 50 developed nausea. One child experienced intractable screaming after administering 2 mg/kg of IM ketamine, and another patient developed visual hallucinations within 24 hours of 5 mg/kg IM ketamine administration.
Lightdale et al., 2011 [16]		United States	37 patients (22 males and 15 females)	Initial 1 mg/kg bolus dose up to a maximum dose of 70 mg followed by 2 additional bolus doses after every 5 minutes	0.05 – 0.3 mg/kg IV midazolam every 3 minutes up to a maximum dose of 15mg followed by 1 – 5 u/kg IV fentanyl every 5 minutes up to a maximum dose of 250u/kg	GI endoscopy	2 cases of laryngospasm were experienced after ketamine administration. One case of oxygen desaturation requiring positive pressure ventilation was recorded after ketamine administration.
Krick et al., 2013 [17]	Observational study	South Africa	110 women (Age 18 to 55 years)	IV 0.25 – 0.5 mg/kg	IV 100mcg fentanyl and IV 5mg midazolam	Uterine evacuation for incomplete miscarriage	Pain scores were significantly lower in the Ketamine group than in the midazolam/fentanyl group ((3.28 (2.41-4.16) vs. 5.93 (5.26-6.60); p < 0.0001, respectively). The side effects recorded after ketamine administration included; 3 cases of objective hallucinations, 4 cases of increased talkativeness, 3 cases of visible distress, and 2 cases of increased salivation/sweating.
Margaret et al., 2021 [18]	RCT	United States	5 patients (2 females and 3 males; mean age 2 + 0.61 years)	3 mg/kg intranasal ketamine (max 100 mg) and 0.03 ml/kg saline (max 2ml)	0.3 mg/kg intranasal midazolam (max 10 mg) followed by 1.5 mcg/kg fentanyl (max 100 mcg).	Laceration repair	The mean pain scores recorded during laceration repair were 4 (4 – 4.5) and 0 (0 – 0.0) in the IN ketamine and IN midazolam/ fentanyl groups, respectively. None of the patients in the two groups died or experienced any adverse events associated with the sedation regimens. The procedure was successful in all patients after receiving the sedation regimens

Pain Scores

**Figure 4:**
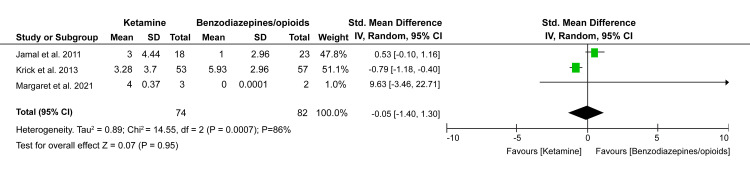
A Forest plot comparing pain scores during procedures between ketamine and midazolam/fentanyl Jamal et al., 2011 [12], Krick et al., 2013 [17], Margaret et al., 2021 [18]

Pain during the procedures was reported in three studies and was measured using the 11-point numeral rating score (NRS). Data pooled from these studies showed that the overall pain score did not differ between the ketamine and midazolam/fentanyl groups (SMD: -0.05; 95% CI: -1.40 - 1.30; p = 0.95). However, the analysis showed substantial heterogeneity, as described in Figure 4.

Sedation Depth

**Figure 5:**
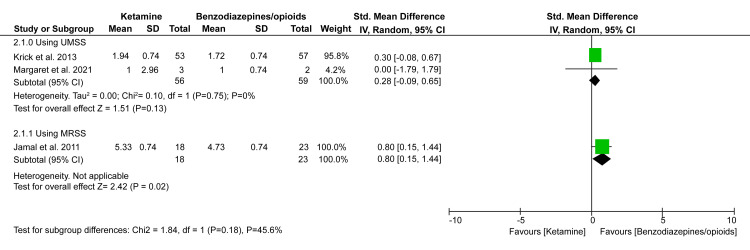
A Forest plot comparing sedation depth between ketamine and midazolam/fentanyl Krick et al., 2013 [17], Margaret et al., 2021 [18], Jamal et al., 2011 [12]

Two studies calculated the overall sedation depth using the UMSS while one used the MRSS. Data pooled from the two studies using the UMSS showed that the mean sedation depth between the groups was similar (SMD: 0.28; 95% CI: -0.09 - 0.65; p = 0.12). However, data from the study using MRSS showed that ketamine offered significantly more profound sedation than the midazolam/fentanyl combination (SMD:0.80; 95% CI: 0.15 - 1.44; p = 0.02) as described in Figure 5.

Adverse Events

**Figure 6:**
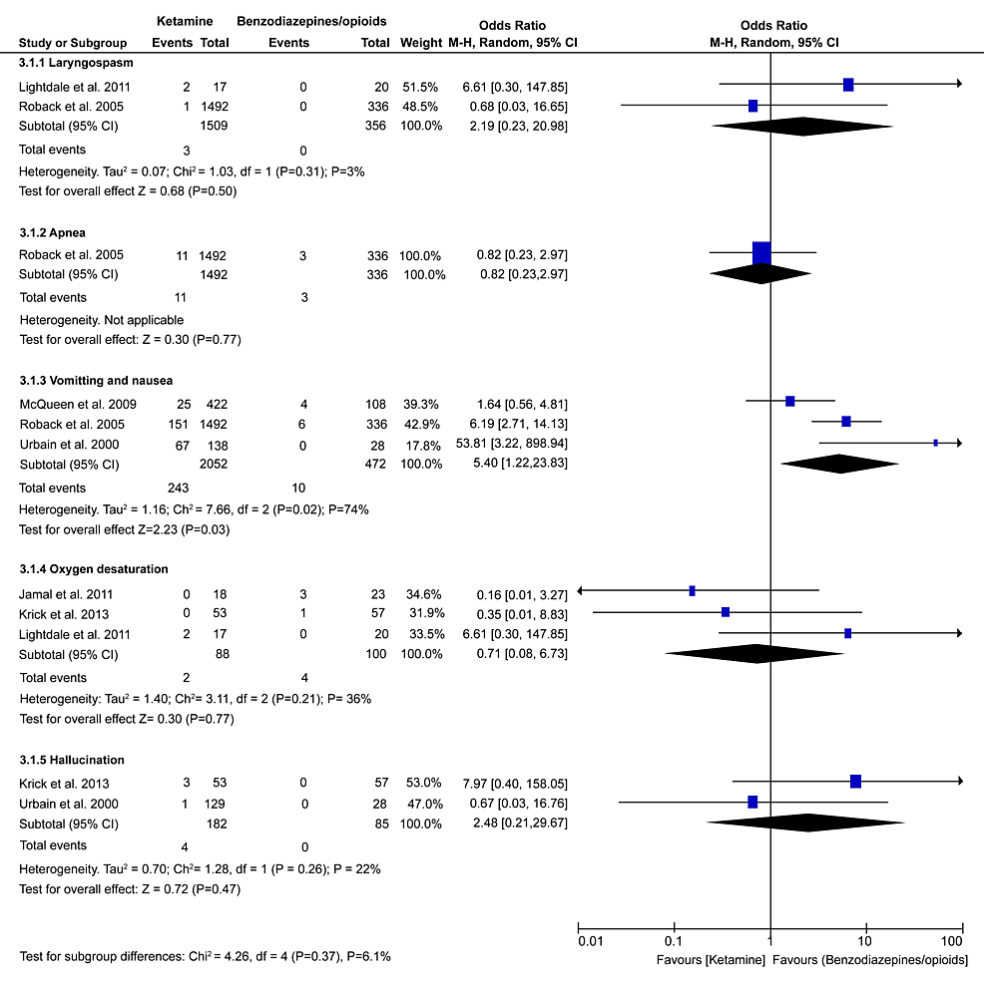
A Forest plot comparing adverse events between ketamine and midazolam/fentanyl Jamal et al., 2011 [12], Roback et al., 2005 [13], McQueen et al., 2009 [14], Urbain et al., 2000 [15], Lightdale et al., 2011 [16], Krick et al., 2013 [17], Margaret et al., 2021 [18]

Adverse events reported in the current study included vomiting and nausea, visual hallucination, laryngospasm, apnea, and oxygen desaturation requiring intervention (Figure 6).

Our meta-analysis revealed that laryngospasm was highly associated with ketamine than midazolam/fentanyl (0.2% vs. 0%). However, the statistical analysis showed that the difference was statistically insignificant (OR: 2.19; 95% CI: 0.23 - 20.98; p = 0.50). Similarly, our meta-analysis did not show any significant difference in the incidence of apnea (OR: 0.82; 95% CI: 0.23 - 2.97; p = 0.77), visual hallucination (OR: 2.48; 95% CI: 0.21 - 29.67; p = 0.47)., and oxygen desaturation (OR: 0.71; 95% CI: 0.08 - 6.73; p = 0.77). However, the frequency of visual hallucination was high when ketamine was used for sedation (2% vs. 0%). In comparison, the frequency of oxygen desaturation was high when midazolam/fentanyl was used for the procedural sedation (4% vs. 2.3%). In addition, our meta-analysis has revealed that ketamine is significantly associated with increased vomiting and nausea than midazolam/fentanyl (OR: 5.40; 95% CI: 1.22 - 22.83; p = 0.03).

Discussion

Procedural sedation is a routine treatment for patients enduring painful and anxiety-provoking procedures in the ED. Enormous inquiry on the safety and efficiency of distinct sedation regimens has been carried out; nonetheless, little is understood about the efficacy of ketamine compared to a combination of benzodiazepines and opioids. Therefore, the primary aim of this study was to compare ketamine with the most common benzodiazepine/opioid combination (midazolam/fentanyl). Our study has shown that ketamine is as effective as midazolam/fentanyl in alleviating procedural pain and providing procedural sedation. However, we noticed that ketamine is significantly associated with the prevalence of vomiting than the midazolam/fentanyl combination.

Although our meta-analysis results suggest that ketamine might be effective as a midazolam/fentanyl combination in providing procedural sedation, contradictory evidence has been reported in a study involving children undergoing gastrointestinal (GI) endoscopy [16]. In that study, the effectiveness of the sedation regimens was carried out using the Ohio State University Behavioral Scale (OSUBS), which is considered in other studies as a reliable and valid method for evaluating patients' behavior during dental and GI endoscopy procedures [19,20]. The study described effective sedation as patients rendered “quiet, still and unrestrained,” of which ketamine was considered more effective than the midazolam/fentanyl combination. On the other hand, ineffective sedation was described as patients who were “vocalizing distress, moving and restrained.” However, it is important to note that this study was subject to various limitations that might have influenced its results. First, the study sample size was small, introducing a selection bias to the study results. Second, the study used a broad definition for “moving” and “needing restraint,” of which movement was defined as either intentional (combative) or unintentional (drug-induced). Based on these limitations, the conclusions made in that study cannot be used to guide clinical care in procedural sedation. More randomized trials are required to establish those results fully.

The other primary aim of this study was to compare the adverse events associated with the sedation regimens. We found that ketamine was significantly associated with an increased risk of vomiting and nausea. This finding was consistent with a recent prospective study of 151 patients, which reported that vomiting and nausea was the second most common adverse event occurring at a rate of 28.7% (25/151) after sedation with ketamine [21]. Similarly, a previous study that commonly used ketamine for procedural sedation reported that vomiting and nausea were the most common adverse events observed in the patients. According to the results presented in that study, adverse events were witnessed in 10 patients, of which six out of the nine patients that received ketamine for procedural sedation vomited [22].

Furthermore, our recent meta-analysis comparing ketofol (a combination of ketamine and propofol) to ketamine alone showed that ketamine was significantly associated with increased nausea and vomiting [23]. All these studies report incidences of vomiting and nausea during the sedation period; however, evidence shows that delayed cases of vomiting and nausea might be observed in patients receiving ketamine, according to McQueen and colleagues. Post-discharge vomiting was recorded in 110 patients that had received ketamine as the agent for procedural sedation. In contrast, none of the patients receiving the midazolam/fentanyl combination had post-discharge vomiting. However, more studies evaluating post-discharge adverse events are required to establish this finding fully.

Our meta-analysis has also confirmed that the prevalence of laryngospasm is higher for patients receiving ketamine than the midazolam/fentanyl combination (0.2% vs. 0%, respectively). The prevalence of laryngospasm recorded in our study aligns with that reported in previous meta-analyses. Green and colleagues pooled data from 32 studies evaluating laryngospasm in patients receiving ketamine and found a prevalence of 0.26% [7]. The prevalence rate recorded in this meta-analysis and ours is low, suggesting that laryngospasm related to ketamine is relatively uncommon and is usually transient and responds quickly to oxygen and assisted ventilation. This finding is evident in a study by Lightdale and colleagues where one patient who developed a case of laryngospasm and an oxygen desaturation of 50% was intervened using positive pressure ventilation and returned to full spontaneous ventilation and oxygen saturation of 100% [16]. Evidence also suggests ketamine can be associated with recurrent laryngospasm in the ED. For example, Cohen and colleagues reported recurrent episodes of laryngospasm in two pediatric patients who received intramuscular ketamine for sedation [24].

Additionally, a case report of a seven-year-old patient receiving intravenous ketamine reported recurrent episodes of laryngospasm [25]. According to this case report, the first episode of laryngospasm occurred even before the procedure, and it was quickly reversed using positive pressure ventilation (PPV). The second episode occurred when the patient was shifted on a transport trolley and was resolved using 5 mg of succinylcholine and PPV. After this episode, three other episodes of laryngospasm happened, and no other episode was recorded.

Our analysis also shows that even though the difference is insignificant, the incidence of hypoxemia (oxygen saturation less than 90%) is higher in the midazolam/fentanyl group than in the ketamine group (4% vs. 2.3%, respectively). The oxygen desaturation observed in patients sedated with the midazolam/fentanyl combination can be attributed to the fact that these agents cause dose-related suppression of the airway protective reflexes and ventilatory drive. Therefore, EPs using these agents for procedural sedation must be comfortable with airway management and well-informed on the pertinent reversal agents. However, it is worth noting that in some of the oxygen desaturation events, the procedures are not suspended since actions such as elevation of the jaw and nasal catheter oxygen flow are carried out to reverse the condition [26].

Patient or clinician satisfaction, adequate sedation, and the success of the procedures are also crucial in assessing the efficacy of the two sedation regimens. A randomized trial that compared intranasal (IN) ketamine with IN midazolam/fentanyl analyzed the satisfaction of nurses and physicians based on the Likert scale and found that the clinicians were more satisfied with the use of IN midazolam/fentanyl than IN ketamine (100% (4/4) vs. 16.7% (1/6), respectively) [18]. However, a Canadian study showed that of the 90 parents reached during the follow-up, 54% were able to provide their response, of which they responded that they were equally satisfied with the procedural sedation provided to their children during painful procedures and would choose similar sedation techniques in the future [15]. Furthermore, the study claimed that 95% of 129 patients receiving ketamine had adequate sedation while midazolam/fentanyl was sufficient for all patients. Similarly, South African research revealed that ketamine and combining fentanyl and midazolam effectively provided appropriate pain management. All operations were completed under procedural sedation without being abandoned to transition to general anesthesia [17]. Jamal and colleagues also showed that the procedures were equally successful in 95.7% of midazolam/fentanyl patients and 94.4 of ketamine patients. If the results of these studies are anything to go by, we can presume that ketamine and midazolam/fentanyl are equally effective in providing procedural sedation. However, it is worth noting that most of these studies are designed as observational studies with low-quality evidence, and further controlled randomized trials are required to confirm their results.

Monitoring changes in vital signs is also crucial in evaluating the efficacy of procedural sedation agents. A study comparing sedation in patients undergoing uterine evacuation for incomplete miscarriage reported a significant rise in systolic blood pressure (SBP) (from 125 mmHg to 130 mmHg, p = 0.026) and diastolic blood pressure (DBP) (from 66 mmHg to 74 mmHg, p = 0.0001) when using ketamine [17]. However, the data did not show any significant change in either DBP or SBP when using a midazolam/fentanyl combination for sedation. The study results also revealed that the sedation regimens did not significantly affect the heart rate. This effect of ketamine to increase SBP and DBP may be advantageous for patients with compensated hemodynamic instability, where a combination of fentanyl and midazolam may result in a breakdown in compensation. However, this effect also means that ketamine should be used sparingly in patients with underlying hypertensive disorders, especially those at a higher risk of cerebrovascular events.

Although we aimed to compare the efficacy and adverse events of ketamine to a combination of midazolam and fentanyl, evidence suggests that dosages and routes of administration have varied effects on these outcomes. A previous large meta-analysis found no association between the standard dosing range with adverse events; however, unusually high IV doses (initial dose >= 2.5mg/kg or a total dose of >= 5.0 mg/kg) was associated with an increased risk for apnea and recovery agitation [7]. Therefore, it is safe to say that there is no benefit of using 1 mg/kg IV rather than 2 mg/kg IV or the use of 3 mg/kg IM instead of 4-5 mg/kg IM, except for the fact that lower doses have a slightly faster recovery [27]. However, recommendations have been made for clinicians to adopt high-dose ketamine because it is consistently less effective in lower doses [28]. In the early 1970s, anesthesiologists used relatively higher doses of ketamine (7-15 mg/kg IM) compared to what is currently being advocated; however, a systematic review has reported no difference in the adverse event profile between the higher doses and the standard dosing [29].

Additionally, studies have shown that IM and IV administration have similar risks for respiratory and air adverse events and recovery agitation [7,30,31]. However, IM administration has been associated with increased vomiting [30,32] and a longer recovery rate [33]. IV administration can be preferred when venous access is rapidly obtained with minimum patient upsets. In contrast, inexpensive and straightforward IM administration can be selected for other settings. Evidence also shows that IV administration can be advantageous in lengthy procedures (> 20 minutes), permitting convenient repeated dosing. Moreover, IV administration has been preferred for adult patients during unpleasant recovery reactions due to the occasional combativeness, and midazolam is provided to alleviate these reactions [34,35].

Current studies have shown that combining ketamine with other sedation regimens may offer safer and more effective procedural sedation than ketamine alone or the midazolam/fentanyl combination. A randomized trial of 260 patients compared the efficacy of ketamine-midazolam with fentanyl-midazolam and reported that patients in the ketamine-midazolam group experienced less pain and anxiety and greater orthopedists’ satisfaction. However, the study reported similar amnesia and sedation depth rates between the groups and recorded a significantly longer recovery time in the ketamine-midazolam group [8]. On the other hand, a double-blind, randomized trial of 141 patients reported that ketamine-midazolam was significantly associated with increased risks of adverse events than fentanyl-midazolam [36]. However, sedation with ketamine-midazolam was associated with reduced durations of hypoxia and pain scores during the reduction procedures. Similarly, a double-blind, randomized trial of 130 patients undergoing procedural sedation for fracture reductions reported a significantly higher risk of adverse events among patients that received the ketamine/midazolam combination than the midazolam/fentanyl combination (16 (51.6%) vs. 2 (6.7%), p < 0.0001). However, the study suggested that the ketamine/midazolam combination can be considered a choice for orthopedic procedures in the ED since it offers a low risk for hypoxia and provides better analgesia by lowering the pain during the procedures [9].

Additionally, a recent randomized trial showed that even though the risk of vomiting was high with ketamine/midazolam, it was significantly associated with a shorter duration for the induction of sedation and offered a less painful procedure than the midazolam/fentanyl combination [37]. Our previous systematic review also deduced that ketamine is more efficient in providing sedation than a combination of ketamine and haloperidol; however, it was associated with a significant risk for hypertension and tachycardia; thus, it should be used while taking into consideration the complications [38]. Moreover, we have previously shown opioids alone may offer better analgesia for patients with hip fractures; however, it is far less superior to ultrasound-guided regional analgesia [39].

Limitations

The interpretation of results provided in the current study should consider several limitations. First, most of the studies included in this systematic review were non-randomized, and some had petite sample sizes, thus limiting the study results. This limitation means that our results can only be improved if more randomized trials are carried out to confirm our results. Second, high heterogeneity values in some pooled effect sizes have been recorded. This heterogeneity can be attributed to the fact that we pooled data utilizing different doses and routes of administration.

Moreover, the effects of routes of administration and dosages on the efficacy and safety of the sedation regimens cannot be established in our results. Third, our results used sedation depth to identify the effectiveness of the sedation regimens and used the MRSS and UMSS only. However, using the OSUBS to evaluate the behavior of patients during the sedation period might have been the best way to identify the effectiveness of these sedation regimens. Moreover, the data for adequate sedation, satisfaction, and successful procedures is essential for evaluating the efficacy of the sedation regimens; but it was limited; thus, we could not carry out a meta-analysis on these outcomes. Finally, the eligibility criteria outlined in this review limited us to studies only published in English. The data from studies written in other languages that would have otherwise improved our results were eliminated.

## Conclusions

Our data suggest that ketamine alone is as effective as a combination of midazolam and fentanyl in providing procedural sedation and analgesia in the ED. However, it is highly associated with vomiting, nausea, visual hallucination, and laryngospasm, meaning it is less safe than the midazolam/fentanyl combination. Nevertheless, evidence shows that ketamine is associated with a significant increase in SBP and DBP; thus, it should be used sparingly in patients exhibiting hypertensive disorder, especially those at a higher risk of cerebrovascular events. If we consider safety, we can recommend using the midazolam/fentanyl combination for procedural sedation in the ED. However, a physician comfortable with airway management should provide this sedation regimen due to high incidences of oxygen desaturation.

## References

[1] Quine MA, Bell GD, McCloy RF, Charlton JE, Devlin HB, Hopkins A (1995). Prospective audit of upper gastrointestinal endoscopy in two regions of England: safety, staffing, and sedation methods. Gut.

[2] Smits GJ, Kuypers MI, Mignot LA (2017). Procedural sedation in the emergency department by Dutch emergency physicians: a prospective multicentre observational study of 1711 adults. Emerg Med J.

[3] Sacchetti A, Senula G, Strickland J (2007). Procedural sedation in the community emergency department: initial results of the ProSCED registry. Acad Emerg Med.

[4] McCarty EC, Mencio GA, Walker LA, Green NE (2000). Ketamine sedation for the reduction of children's fractures in the emergency department. J Bone Joint Surg Am.

[5] Green SM, Roback MG, Kennedy RM, Krauss B (2011). Clinical practice guideline for emergency department ketamine dissociative sedation: 2011 update. Ann Emerg Med.

[6] Eberson CP, Hsu RY, Borenstein TR (2015). Procedural sedation in the emergency department. J Am Acad Orthop Surg.

[7] Green SM, Roback MG, Krauss B (2009). Predictors of airway and respiratory adverse events with ketamine sedation in the emergency department: an individual-patient data meta-analysis of 8,282 children. Ann Emerg Med.

[8] Kennedy RM, Porter FL, Miller JP, Jaffe DM (1998). Comparison of fentanyl/midazolam with ketamine/midazolam for pediatric orthopedic emergencies. Pediatrics.

[9] Cevik E, Bilgic S, Kilic E, Cinar O, Hasman H, Acar AY, Eroglu M (2013). Comparison of ketamine-low-dose midozolam with midazolam-fentanyl for orthopedic emergencies: a double-blind randomized trial. Am J Emerg Med.

[10] Malviya S, Voepel-Lewis T, Tait AR, Merkel S, Tremper K, Naughton N (2002). Depth of sedation in children undergoing computed tomography: validity and reliability of the University of Michigan Sedation Scale (UMSS). Br J Anaesth.

[11] Sheahan CG, Mathews DM (2014). Monitoring and delivery of sedation. Br J Anaesth.

[12] Jamal SM, Fathil SM, Nidzwani MM, Ismail AK, Yatim FM (2011). Intravenous ketamine is as effective as midazolam/fentanyl for procedural sedation and analgesia in the emergency department. Med J Malaysia.

[13] Roback MG, Wathen JE, Bajaj L, Bothner JP (2005). Adverse events associated with procedural sedation and analgesia in a pediatric emergency department: a comparison of common parenteral drugs. Acad Emerg Med.

[14] McQueen A, Wright RO, Kido MM, Kaye E, Krauss B (2009). Procedural sedation and analgesia outcomes in children after discharge from the emergency department: ketamine versus fentanyl/midazolam. Ann Emerg Med.

[15] Ip U, Saincher A (2000). Safety of pediatric procedural sedation in a Canadian emergency department. CJEM.

[16] Lightdale JR, Mitchell PD, Fredette ME, Mahoney LB, Zgleszewski SE, Scharff L, Fox VL (2011). A pilot study of ketamine versus midazolam/fentanyl sedation in children undergoing GI endoscopy. Int J Pediatr.

[17] Krick D (2013). Study of efficacy of ketamine analgesia for surgical management of incomplete miscarriages. https://open.uct.ac.za/handle/11427/3047.

[18] Menoch MJ (2018). Comparison of sedation, pain, and care provider satisfaction between the use of intranasal ketamine versus intranasal midazolam and fentanyl during laceration repair. clinicaltrials.gov.

[19] Lochary ME, Wilson S, Griffen AL, Coury DL (1993). Temperament as a predictor of behavior for conscious sedation in dentistry. Pediatr Dent.

[20] McCann W, Wilson S, Larsen P, Stehle B (1996). The effects of nitrous oxide on behavior and physiological parameters during conscious sedation with a moderate dose of chloral hydrate and hydroxyzine. Pediatr Dent.

[21] Dilip TS, Chandy GM, Hazra D, Selvan J, Ganesan P (2021). The adverse effects of ketamine on procedural sedation and analgesia (PSA) in the emergency department. J Family Med Prim Care.

[22] Salleeh HM, Ahmadi TA, Mujawar Q (2014). Procedural sedation for pediatric patients in the emergency department at King Khalid University Hospital, Riyadh, K.S.A. J Emerg Trauma Shock.

[23] Zaki HA, Shallik N, Shaban E (2022). An analytical comparison between ketamine alone and a combination of ketamine and propofol (ketofol) for procedural sedation and analgesia from an emergency perspective: a systematic review and meta-analysis. Cureus.

[24] Cohen VG, Krauss B (2006). Recurrent episodes of intractable laryngospasm during dissociative sedation with intramuscular ketamine. Pediatr Emerg Care.

[25] Baduni N, Sanwal MK, Jain A, Kachru N (2010). Recurrent episodes of intractable laryngospasm followed by laryngeal and pulmonary oedema during dissociative anaesthesia with intravenous ketamine. Indian J Anaesth.

[26] Qian H, Sujuan Z, Jun Z (2020). Safety and efficacy of fentanyl combined with midazolam in bronchoscopy under bispectral index-guided conscious sedation [Preview]. Research Square.

[27] Shah A, Mosdossy G, McLeod S, Lehnhardt K, Peddle M, Rieder M (2011). A blinded, randomized controlled trial to evaluate ketamine/propofol versus ketamine alone for procedural sedation in children. Ann Emerg Med.

[28] Green SM, Hummel CB, Wittlake WA, Rothrock SG, Hopkins GA, Garrett W (1999). What is the optimal dose of intramuscular ketamine for pediatric sedation?. Acad Emerg Med.

[29] Green SM, Johnson NE (1990). Ketamine sedation for pediatric procedures: part 2, review and implications. Ann Emerg Med.

[30] Green SM, Roback MG, Krauss B (2009). Predictors of emesis and recovery agitation with emergency department ketamine sedation: an individual-patient data meta-analysis of 8,282 children. Ann Emerg Med.

[31] Sener S, Eken C, Schultz CH, Serinken M, Ozsarac M (2011). Ketamine with and without midazolam for emergency department sedation in adults: a randomized controlled trial. Ann Emerg Med.

[32] Thorp AW, Brown L, Green SM (2009). Ketamine-associated vomiting. Is it dose-related?. Pediatr Emerg Care.

[33] Green SM, Denmark TK, Cline J, Roghair C, Abd Allah S, Rothrock SG (2001). Ketamine sedation for pediatric critical care procedures. Pediatr Emerg Care.

[34] Green SM, Li J (2000). Ketamine in adults: what emergency physicians need to know about patient selection and emergence reactions. Acad Emerg Med.

[35] Green SM, Clem KJ, Rothrock SG (1996). Ketamine safety profile in the developing world: survey of practitioners. Acad Emerg Med.

[36] Abdolrazaghnejad A, Banaie M (2017). Fentanyl-midazolam vs. midazolam-ketamine regarding patient sedation analgesia for emergency orthopedic procedures. Bangladesh J Pharmacol.

[37] Monsereenusorn C, Malaithong W, Lertvivatpong N, Photia A, Rujkijyanont P, Traivaree C (2022). The efficacy and safety of midazolam with fentanyl versus midazolam with ketamine for bedside invasive procedural sedation in pediatric oncology patients: a randomized, double-blinded, crossover trial. Pediatr Hematol Oncol.

[38] Zaki HA, Shaban E, Bashir K, Iftikhar H, Zahran A, Salem EE, Elmoheen A (2022). A comparative analysis between ketamine versus combination of midazolam and haloperidol for rapid safe control of agitated patients in emergency department: a systematic review. Cureus.

[39] Zaki HA, Iftikhar H, Shallik N, Elmoheen A, Bashir K, Shaban EE, Azad AM (2022). An integrative comparative study between ultrasound-guided regional anesthesia versus parenteral opioids alone for analgesia in emergency department patients with hip fractures: a systematic review and meta-analysis. Heliyon.

